# The *Yersinia* Type III secretion effector YopM Is an E3 ubiquitin ligase that induced necrotic cell death by targeting NLRP3

**DOI:** 10.1038/cddis.2016.413

**Published:** 2016-12-08

**Authors:** Congwen Wei, Ying Wang, Zongmin Du, Kai Guan, Ye Cao, Huiying Yang, Pengyu Zhou, Feixiang Wu, Jiankang Chen, Penghao Wang, Zirui Zheng, Pingping Zhang, Yanhong Zhang, Shengli Ma, Ruifu Yang, Hui Zhong, Xiang He

**Affiliations:** 1 State key Laboratory of Pathogen and Biosecurity, Beijing Institute of Biotechnology, Beijing, China; 2 State key Laboratory of Pathogen and Biosecurity, Beijing Institute of Microbiology and Epidemiology, Beijing, China; 3 The General Hospital of Chinese People's Armed Police Forces, Beijing, China; 4 Department of Hepatobiliary Surgery, Affiliated Tumor Hospital of Guangxi Medical University, Nanning, China

## Abstract

*Yersinia pestis* uses type III effector proteins to target eukaryotic signaling systems. The Yersinia outer protein (Yop) M effector from the *Y. pestis* strain is a critical virulence determinant; however, its role in *Y. pestis* pathogenesis is just beginning to emerge. Here we first identify YopM as the structural mimic of the bacterial IpaH E3 ligase family *in vitro*, and establish that the conserved CLD motif in its N-terminal is responsible for the E3 ligase function. Furthermore, we show that NLRP3 is a novel target of the YopM protein. Specially, YopM associates with NLRP3, and its CLD ligase motif mediates the activating K63-linked ubiquitylation of NLRP3; as a result, YopM modulates NLRP3-mediated cell necrosis. Mutation of YopM E3 ligase motif dramatically reduces the ability of *Y. pestis* to induce HMGB1 release and cell necrosis, which ultimately contributes to bacterial virulence. In conclusion, this study has identified a previously unrecognized role for YopM E3 ligase activity in the regulation of host cell necrosis and plague pathogenesis.

Inflammatory reactions in response to pathogen infection are orchestrated by complex soluble mediators, including locally released cytokines and chemokines, such as IL-1*β* and IL-18.^1, 2, 3, 4^ The activation of caspase-1 is essential for the processing of pro-IL-1*β* and pro-IL-18 and the secretion of their mature biologically active forms.^5^ A critical step in the activation of caspase-1 is the assembly of a large protein complex (inflammasome) containing the NOD-like receptors (NLRs), adapter ASC, and pro-caspase-1.^6^ The inflammasome recognizes intracellular damage-associated molecular patterns (DAMPs) derived from pathogens by cytosolic NLRs.^7^ NLRs undergo oligomerization in response to DAMP recognition and recruit ASC by PYD-PYD interaction.^8, 9^ Subsequently, pro-caspase-1 is recruited through the C-terminal caspase recruitment domain (CARD) of ASC, which is essential for its activation.^10, 11, 12, 13^

The best-characterized inflammasome is the NOD-like receptor (NLR)-family pyrin domain-containing 3 (NLRP3, also known as Nalp3 or cryopyrin) inflammasome, which can be activated by a wide range of stimuli.^14, 15, 16, 17, 18^ Two different types of NLRP3-dependent cell death have been reported.^19^ Pyroptosis is an inflammatory program of cell death, which is caspase-1-dependent.^10^ Activation of pyroptosis promotes innate immune responses, protects against numerous pathogens, and enables clearance of invading pathogens. Therefore, evasion of NLRP3-induced pyroptosis is an important strategy of pathogen.^20, 21^ For example, Kaposi's sarcoma-associated herpes virus encodes an NLRP1 homolog that lacks PYD and CARD domain, which can competently interact with host NLRP3 and inhibits virally induced pyroptosis.^22^

Recent works have implicated the role of NLRP3 in triggering necrosis, which is another form of pathogen-induced cell death independent of caspase-1. Although necrosis induction eliminates pathogen by removal of infected cells, it also leads to exacerbated inflammation and sepsis. In necrotic cells, HMGB1 is passively released into the cytoplasm and extracellular space, where it serves as a useful necrosis indicator and late proinflammatory molecule. HMGB1 has also been suggested to act as an amplifier of neutrophil recruitment, injury amplification, and host lethality.^23^ For example, HMGB1 is released during dengue hemorrhagic fever and dengue shock syndrome, impacting on disease pathogenesis.^24^ *Shigella flexneri* causes NLRP3-dependent necrosis and HMGB1 release in macrophages associated with further tissue damage.^25^ *Yersinia pestis* infection can also induce macrophage necrosis, which is dispensable for innate host resistance but contributes to enhanced cytotoxicity.^26^ However, the role of NLRP3-dependent necrosis in *Y. pestis* pathogenesis remains further investigation.

YopMs, one of the T3SS effectors of *pestis*, are composed of a variable number of 20 or 22 amino acid (aa) leucine-rich repeats (LRRs), which are known to mediate protein–protein interactions.^27, 28, 29, 30^ YopM has a nuclear localization signal in its C-terminus, and is translocated into the nucleus via an endocytic pathway.^31, 32, 33^ The first identified targets of YopM were ribosomal S6 protein kinase 1 (RSK1) and protein kinase C-like 2 (PRK2).^34^ LRR6 to LRR15 region of YopM is required for PRK2 binding, while C-terminal domain (299-409 aa) is required for binding to RSK1.^35^ Deletion of either of these domains from YopM abrogates the virulence of *Y. pseudotuberculosis via* the orogastric route of infection.^36^ One recent report indicated that YopM is a potent antagonist of both caspase-1 activity and activation.^37^ While all other Yops have been shown to possess enzymatic activity, the YopM protein is believed to be devoid of catalytic activity.

Here we demonstrate that YopM is an E3 ubiquitin ligase with a novel enzyme active site in its N-terminal, which can regulate NLRP3 expression. The conserved CLD motif in the N-terminal of YopM mediates the activating K63-linked ubiquitylation of NLRP3. Mutation of YopM E3 ligase motif (C68A mutant) not only abolishes YopM-induced NLRP3 stabilization but also dramatically reduces the ability of *Y. pestis* to stimulate NLRP3-mediated HMGB1 release and cell necrosis. Thus our results identify a previously unrecognized role for YopM in the regulation of NLRP3-mediated necrosis signaling and plague pathogenesis.

## Results

### *Y. pestis* T3SS YopM is an E3 ubiquitin ligase

**Figure 1:**
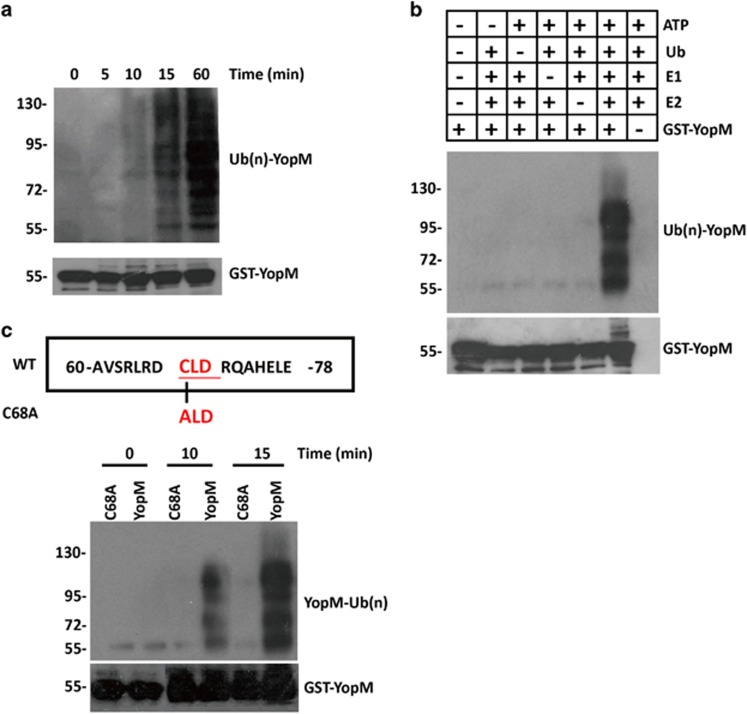
*Y. pestis* T3SS YopM is an E3 ubiquitin ligase. (**a** and **b**) Immunoblotting showing autoubiquitination by GST-YopM in the presence (**a**) or absence (**b**) of E1 and E2, ATP, and Ub. (**c**) Effects of mutations of Cys 68 on YopM–catalyzed polyubiquition chain synthesis. Ubiquitination assays were carried out using GST-YopM and GST-YopM C68A. All cell-based experiments were performed independently two to three times with comparable results

Recent study reported that a CXD catalytic motif in the bacterial pathogen *Shigella flexneri* could function as an E3 ligase.^38^ Subsequent analysis of the amino acid sequences of YopM revealed a conserved CLD motif (C68/L69/D70) in the N-terminal domain, which we hypothesized may function as an novel E3 catalytic domain (Supplementary Table S1). To test this hypothesis, a purified GST-YopM fusion protein was used for autoubiquitination activity assay in the presence of ATP, ubiquitin, E1 and E2 *in vitro*. We detected molecular species 7 kDa larger than corresponding YopM fragments within 10 min of the ubiquitination reaction, and larger species accumulated thereafter (Figure 1a). In accordance with E3 ligase activity, no such species were detected even after 1.5 h of incubation if the reaction lacked ATP, ubiquitin, E1, E2 or YopM (Figure 1b). To better understand the ubiquitin-ligase activity of YopM protein, we created a cDNA construct to express a YopM protein with C68A mutation. In contrast to wild-type (WT) YopM, C68A mutant failed to induce any autoubiquitination (Figure 1c), demonstrating that Cys 68 is the novel active site of YopM for the E3 ubiquitin ligase activity.

### YopM protein associates with NLRP3

**Figure 2:**
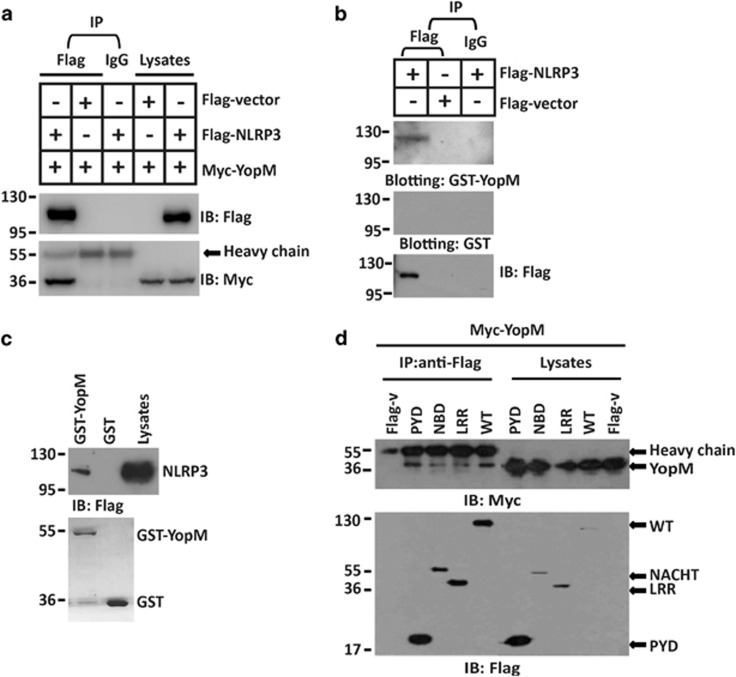
YopM protein associates with NLRP3. (**a**) HEK293 cells were co-transfected with Flag-NLRP3 and Myc-YopM expression plasmids or Flag vector, and anti-Flag M2 Affinity Gel or IgG agarose immunoprecipitates were analyzed by immunoblotting with anti-Myc or anti-Flag antibody. (**b**) Anti-Flag or IgG immunoprecipitates prepared from cells transfected with Flag-NLRP3 or Flag vector-expressing plasmids were subjected to SDS-PAGE and blotted onto nitrocellulose membrane. The nitrocellulose membrane was incubated with soluble GST-YopM (upper) or GST (middle) for 2 h and then analyzed with anti-GST or anti-Flag antibody. (**c**) GST-tagged YopM were subjected to pull-down assay with the lysates of HEK293 cells transfected with Flag-NLRP3 expressing plasmid. Immunoblotting analyses with anti-Flag antibody was shown in the top. Loading of the GST proteins assessed by Coomassie blue staining was shown in the bottom. GST was used as a negative control. (**d**) HEK293 cells were co-transfected with the indicated vector encoding Myc-tagged YopM, Flag-tagged full-length NLRP3 or truncations of NLRP3 containing only residues 1–90 (pyrin domain) (PYD), 91–710 (nucleotide-binding domain) (NBD) or 711–1033 (LRR domain) (LRR), and anti-Flag immunoprecipitates were analyzed by immunoblotting with anti-Myc or anti-Flag antibody. All co-immunoprecipitation experiments were performed independently three times with comparable results. All cell-based experiments were performed independently three times with comparable results

We then explored the possible host target of YopM. Yeast two-hybrid (Y2H) screening data revealed a possible interaction between YopM and NLRP3 (Supplementary Figure S1A). To confirm the yeast two-hybrid analysis, Flag-tagged NLRP3 and Myc-tagged YopM were transfected into HEK293 cells, and a co-immunoprecipitation experiment was performed. Myc-tagged YopM was detected in the anti-Flag immunoprecipitation from cells co-transfected with Flag-NLRP3, but not with a negative control Flag-tagged protein (Figure 2a). Vice versa, Flag-tagged NLRP3 was detected in the anti-Myc immunoprecipitation from cells co-transfected with Myc-YopM, but not with a negative control Myc-tagged protein (Supplementary Figure S1B). To investigate the interaction between YopM variants of other Yersinia strains and NLRP3, Flag-tagged NLRP3 and Myc-tagged YopM (KIM5) were transfected into HEK293 cells, and a co-immunoprecipitation experiment was performed. Myc-tagged YopM (KIM5) was also detected in the anti-Flag immunoprecipitation from cells co-transfected with Flag-NLRP3 (Supplementary Figure S1C). To demonstrate the interaction of YopM and NLRP3 *in vitro*, lysates from HEK293 cells expressing Flag-NLRP3 were incubated with glutathione S-transferase (GST) or GST-YopM fusion protein. Analysis of the absorbates by immunoblotting with anti-GST showed that NLRP3 bound GST-YopM, but not GST (Figure 2b). GST pull-down assay also showed an *in vitro* interaction between YopM and NLRP3 (Figure 2c). NLPR3 consists of an N-terminal pyrin domain (PYD), a central nucleotide binding or oligomerization domain (NBD, NOD, or NACHT) and C-terminal LRRs. We then further analyzed the ability of the three domains of NLRP3 to associate with YopM. To this end, all three domains were involved in the interaction with YopM (Figure 2d). Since NLRP3 interacted with YopM, we suggested that NLRP3 might be a potential substrate of YopM E3 ubiquitin ligase.

### YopM mediates K63-linked ubiquitination of NLRP3

**Figure 3:**
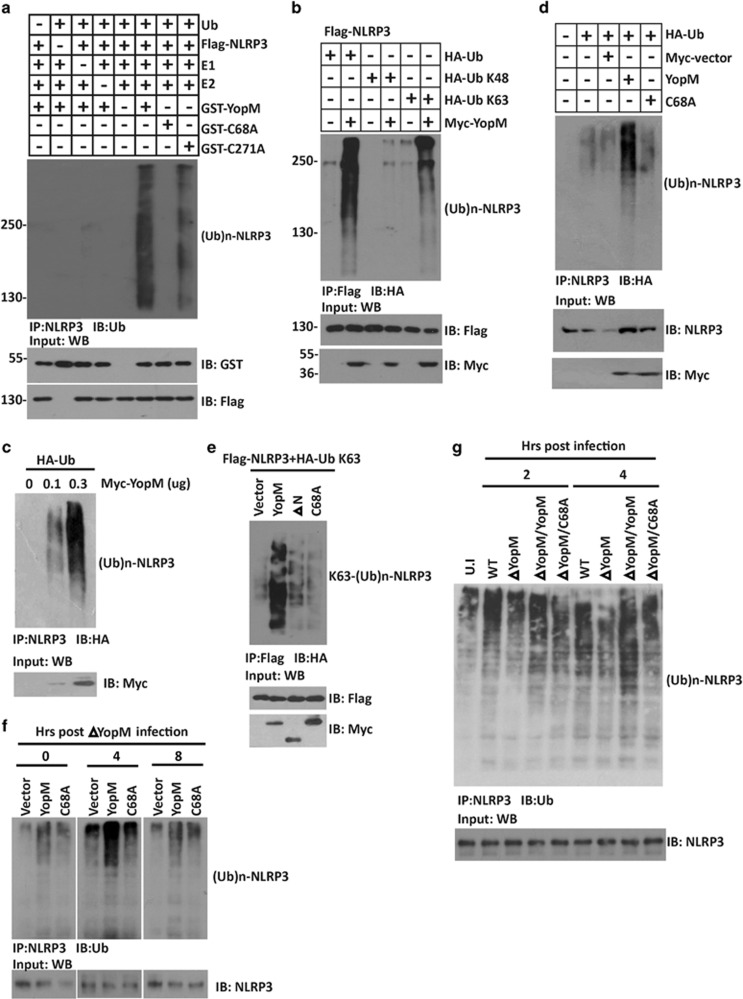
YopM mediates K63-linked ubiquitination of NLRP3. (**a**) *In vitro* ubiquitylation assay with Flag-NLRP3 and a mixture of E1, E2s, ATP, ubiquitin, GST-YopM, or GST-YopM C68A. GST-YopM and Flag-NLRP3 were used to show equal loading. (**b**) Immunoblotting showing ubiquitination levels of NLRP3 in HEK293 cells transfected with Flag-NLRP3, Myc-YopM, and HA-tagged ubiquitin, HA-tagged K48 ubiquitin (Ub K48) or HA-tagged K63 ubiquitin (Ub K63), treated with MG132 (10 *μ*M) for 12 h. (**c**) Immunoblotting showing ubiquitination levels of endogenous NLRP3 in HEK293 cells transfected with HA-tagged ubiquitin together with different doses of Myc-YopM. (**d**) Immunoblotting showing ubiquitination levels of endogenous NLRP3 in BMDMs transfected with HA-tagged ubiquitin together with Myc-YopM or Myc-YopM C68A. (**e**) Immunoblotting showing ubiquitination level of NLRP3 in HEK293 cells transfected with Flag-NLRP3, HA-tagged K63 ubiquitin together with Myc-YopM, Myc-YopM C68A, or Myc-YopM ΔN. (**f**) BMDMs cells transfected with Flag-YopM or Flag-YopM C68A were infected *Y. pestis* ΔYopM (MOI=20). Cell extracts were prepared at the indicated time points and anti-NLRP3 immunoprecipitates were analyzed by immunoblotting with anti-Ub antibody. (**g**) BMDMs were infected with different background of *Y. pestis*. After infection, cell extracts were prepared at the indicated time points and anti-NLRP3 immunoprecipitates were analyzed by immunoblotting with anti-Ub antibody. Cell-based studies were performed independently three times with comparable results

To explore whether NLRP3 is a substrate of YopM E3 ubiquitin ligase, we performed the *in vitro* ubiquitination assay using Flag-NLRP3 as the substrate. Although we did not find CED motif in the C-terminal tail of YopM, we still mutated the only Cys 271 present in the LRR region and used YopM C271A as a control. Both WT YopM and YopM C271A mutant dramatically enhanced the ubiquitination of NLRP3 in the presence of E1 and E2 (Figure 3a), while YopM C68A mutant failed to increase the ubiquitination level of NLRP3, indicating the importance of Cys 68 active site in regulating NLRP3 functions. Furthermore, immunoprecipitation assay showed that YopM catalyzed the ubiquitination of NLRP3 mainly with ubiquitin containing a lysine residue only at position 63 (all others substituted with arginine), but at very low level with ubiquitin containing a lysine residue only at position 48 (Figure 3b). Congruence results showed that overexpression of YopM led to a considerable increase of endogenous NLRP3 ubiquitination (Figure 3c). In contrast to WT YopM, C68A mutant or ΔN mutant failed to induce any endogenous NLRP3 polyubiquitination (Figures 3d and e). In consistence, the ubiquitination of NLRP3 stimulated by ΔYopM bacterial infection was greatly reduced in the cells expressing YopM C68A than YopM WT (Figure 3f). Similarly, BMDMs infected with WT and ΔYopM/YopM *Y. pesitis*, but not ΔYopM or ΔYopM/C68A, showed increased NLRP3 ubiquitination (Figure 3g). Taken together, these results suggest that the CLD motif (68–70 aa) of YopM is responsible for the E3 catalytic activity of YopM on NLRP3.

### YopM mediates the stabilization of NLRP3

**Figure 4:**
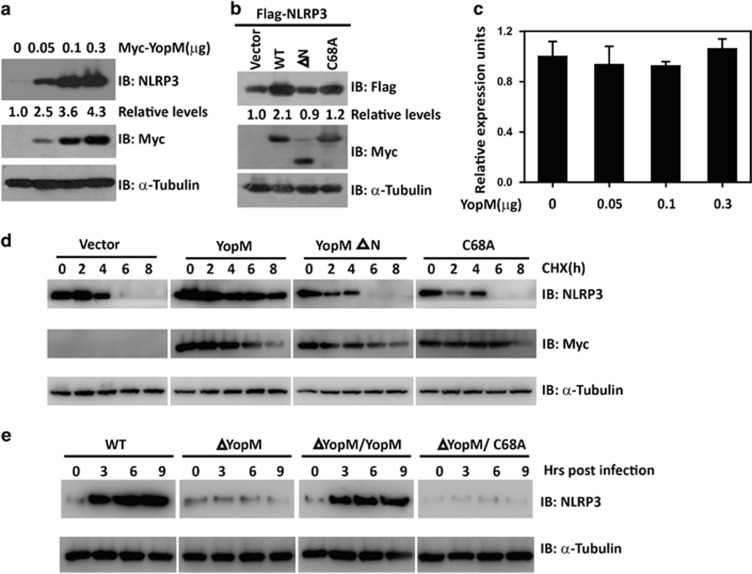
YopM mediates the stabilization of NLRP3. (**a**) Immunoblotting analysis of extracts of HEK293 cells transfected with increasing doses of plasmid for YopM using anti-NLRP3 antibody. *α*-Tubulin was used as equal loading control. (**b**) Immunoblotting analysis of extracts of HEK293 cells transfected with plasmid for Flag-NLRP3 together with Myc-YopM, Myc-YopM ΔN, or Myc-YopM C68A showing NLRP3 stability. *α*-Tubulin was used as equal loading control. (**c**) Quantitative RT-PCR analysis of NLRP3 mRNA levels in HEK293 cells transfected with increasing doses of plasmid for YopM. (**d**) HEK293 cells were transfected with the expression vector encoding Flag-NLRP3, Myc-YopM, Myc-YopM C68A or Myc-YopM ΔN. After 24 h, cells were treated with cycloheximide (10 *μ*M) at the indicated time points, and the level of NLRP3 was monitored by immunoblotting using anti-Flag antibody. *α*-Tubulin was used as equal loading control. (**e**) BMDMs were infected with different background of *Y. pestis*. After infection, cell extracts were prepared at the indicated time points and subjected to immunoblotting. Cell-based studies were performed independently two to three times with comparable results

We next sought to determine the effect of YopM on NLRP3 expression. When HEK293 cells were transfected with increasing amounts of plasmid encoding YopM, we found that the concentration of endogenous NLRP3 protein increased considerably with increasing YopM expression (Figure 4a). However, the YopM E3 ligase activity was required, since YopM C68A mutant lacking E3 ligase activity had compromised activity on NLRP3 stabilization. In contrast to WT YopM, YopM ΔN mutant failed to induce any endogenous NLRP3 stabilization (Figure 4b). Quantitative RT-PCR revealed that YopM overexpression did not change NLRP3 mRNA levels (Figure 4c), suggesting that YopM upregulates NLRP3 protein expression by post-transcriptional mechanism. To determine the specificity of the YopM-mediated stabilization of NLRP3, we did similar experiments with cells expressing TBK1, ASC, pro-caspase-1, NLRP4, or NLRP5 with increasing YopM expression and found that YopM did not affect the concentration of all the other molecules tested (Supplementary Figure S2A). Indeed, the estimated half-life of NLRP3 in the presence of YopM was significantly longer than that in the presence of YopM C68A or YopM ΔN mutant (Figure 4d). We then assessed whether YopM is able to mediate the stabilization of endogenous NLRP3 under physiological conditions by analysis of NLRP3 protein kinetics in response to bacterial infection, and observed that NLRP3 protein levels increased in WT *Y. pestis*-infected BMDMs within 3 h post infection. However, bacteria lacking YopM failed to induce any NLRP3 stabilization (Figure 4e). Moreover, *Y. pestis* infection-induced NLRP3 stabilization also required YopM E3 ligase activity (Figure 4e). These results indicate that NLRP3 is upregulated under physiological conditions of *Y. pestis* infection in a YopM-dependent manner.

### YopM induces NLRP3-dependent necrosis *in vitro*

**Figure 5:**
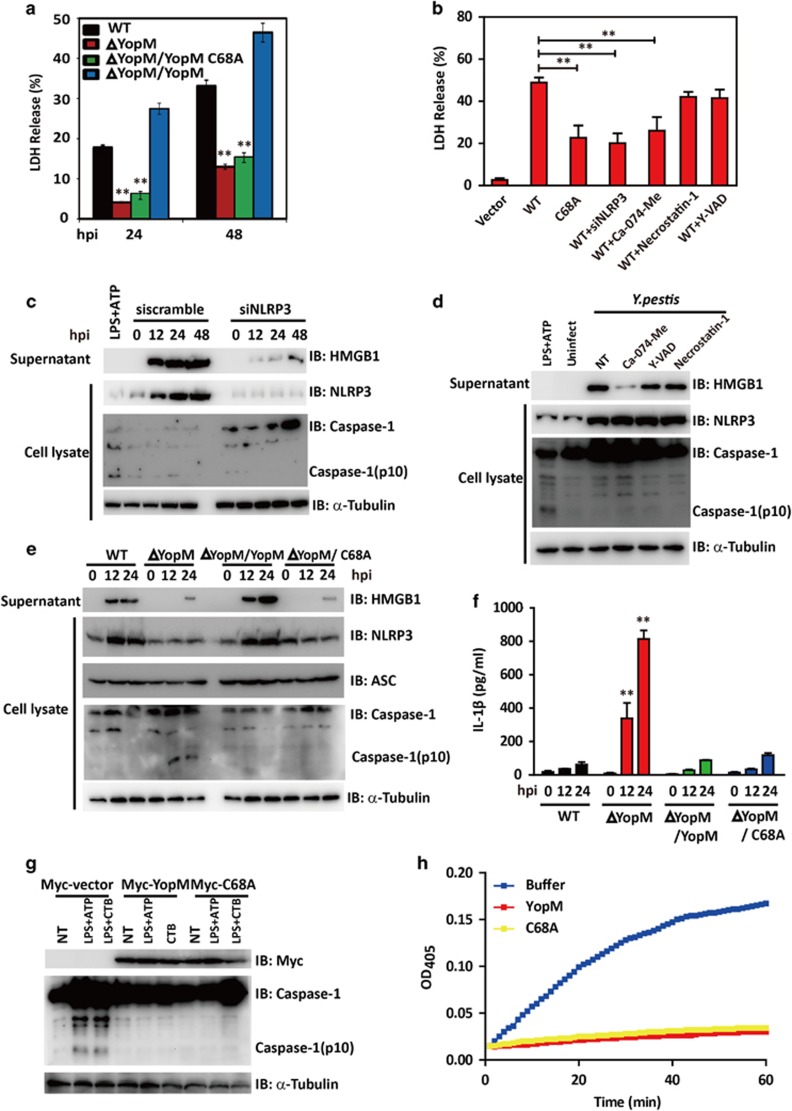
The importance of YopM E3 ligase activity in NLRP3-mediated necrotic cell death. (**a**) BMDMs were infected, respectively, with *Y. pestis* WT, ΔYopM, ΔYopM/YopM or ΔYopM/YopM C68A mutant bacteria for the indicated time (MOI=20). After *Y. pestis* invasion, supernatants from macrophages were collected at the time points indicated, and percent LDH was determined. (**b**) BMDMs cells were treated with or without 100 *μ*M caspase-1-specific inhibitor YVAD-CHO, 50 *μ*M Cathepsin B-specific inhibitor Ca-074-Me or 30 *μ*M RIP1-specific inhibitor necrostatin-1 and then transfected with expression plasmids encoding for Flag-YopM or its mutants or NRLP3 siRNA oligos (siNRLP3). Supernatants were collected at 24 h after transfection and analyzed for LDH release. (**c**) BMDMs cells were transfected with scrambled or NLRP3 siRNA oligos (si NLRP3) and then infected with *Y. pestis*. Cell extracts and supernatants were prepared at indicated time points. The whole-cell lysates were analyzed by immunoblotting with anti-NLRP3, anti-caspase-1 Abs. The supernatants were analyzed by immunoblotting with anti-HMGB1. *α*-Tubulin was used as equal loading control. (**d**) BMDMs cells were treated with or without 100 *μ*M caspase-1-specific inhibitor YVAD-CHO, 50 *μ*M Cathepsin B-specific inhibitor Ca-074-Me or 30 *μ*M RIP1-specific inhibitor necrostatin-1 before infection. BMDMs were infected with *Y. pestis*, cell extracts and supernatants were prepared at 24 h. The whole-cell lysates were analyzed by immunoblotting with anti-NLRP3, anti-caspase-1 Abs. The supernatants were analyzed by immunoblotting with anti-HMGB1. *α*-Tubulin was used as equal loading control. (**e** and **f**) BMDMs were infected, respectively, with *Y. pestis* WT, ΔYopM, ΔYopM/YopM, or ΔYopM/YopM C68A mutant bacteria for the indicated time (MOI=20). After *Y. pestis* invasion, cell extracts and supernatants were prepared at indicated time points. The whole-cell lysates were analyzed by immunoblotting with anti-NLRP3, anti-ASC, and anti-caspase-1 Abs. The supernatants were analyzed by immunoblotting with anti-HMGB1 Abs (**e**) or analyzed by ELISA for IL-1*β* (**f**).*α*-Tubulin was used as equal loading control. (**g**) BMDMs cells were transfected with the expression vector encoding Myc-YopM, Myc-YopM C68A. After 24 h, cells were primed for 4 h with 1 ng/ml LPS and stimulated with ATP (2.5 mM) for 30 min or CTB (20 mg/ml) for 16 h. The whole-cell lysates were analyzed by immunoblotting with anti-Myc and anti-caspase-1 Abs. *α*-Tubulin was used as equal loading control. (**h**) Recombinant caspase-1 was preincubated with GST-YopM or GST-YopM(C68A) for 5 min at 37 °C in assay buffer. Caspase-1 substrate Ac-YVAD-pNA was added, and cleavage was detected by monitoring of absorbance at 405 nm. Cell-based studies were performed at least three times independently with comparable results. Data were presented as mean±S.E.M. Student's *t*-test was used for statistical analysis: **P<0.01

A role for NLRP3 in caspase-1 activation is well studied. Recent works have implicated another role of NLRP3 in triggering macrophages necrosis, which is independent of caspase-1 and IL-1*β.*^39^ We then try to determine whether YopM plays a role in NLRP3-mediated necrosis. We found that infection of WT *Y. pestis* dramatically decreased cell viability in BMDMs, while bacteria lacking YopM (ΔYopM) induced significantly less cell death as measured by lactate dehydrogenase (LDH) release. In addition, YopM-deficient bacteria complemented with YopM C68A induced significantly reduced cell death than those complemented with WT YopM (Figure 5a). Consistent with these findings, ectopic WT YopM expression, but not YopM C68A, led to significant cell death as compared with the vector-transfected control cells (Figure 5b). To further discriminate apoptotic and necrotic cell death induced by *Y. pestis* infection, FACS with Annexin V and PI double staining was performed. H_2_O_2_ was used a positive control to induce cell necrosis death. As was shown in Supplementary Figure S3A, a marked increase of necrosis was detected in WT and ΔYopM/YopM-infected BMDMs compared with that of ΔYopM or ΔYopM/C68A.

HMGB1 release is another distinctive marker to differentiate between necrosis and pyroptosis. *Y. pestis* infection or YopM transfection triggered significant HMGB1 release (Figure 5c and Supplementary Figure S3B), while knocking down of NLRP3 blocked YopM-induced cell death and decreased HMGB1 release (Figures 5b and c), indicating that YopM-induced necrosis is NLRP3-dependent. Previous reports indicated that NLRP3-mediated HMGB1 release is dependent on cathepsin B,^40^ we next sought to determine the effect of caspase-1-specific inhibitor YVAD-CHO, cathepsin B-specific inhibitor Ca-074-Me, and RIP1-specific inhibitor necrostatin-1 on YopM-induced cell necrosis. Pretreatment of cells with YVAD-CHO or necrostatin-1 failed to abrogate necrosis and HMGB1 release induced by YopM overexpression (Figure 5b and Supplementary Figure S3B) or *Y. pestis* infection (Figure 5d and Supplementary Figure S3C). However, Ca-074-Me significantly attenuated LDH release and HMGB1 release in both models, indicating the involvement of cathepsin B in this biological process.

Interestingly, ΔYopM infection in BMDMs failed to upregulate NLRP3 and HMGB1 secretion, but significantly induced caspase-1 activation and IL-1*β* secretion. On the contrary, ΔYopM/YopM rescue strain infection triggered increased HMGB1 release without inducing caspase-1 cleavage and IL-1*β* secretion (Figures 5e and f). We thus concluded that YopM induced cell necrosis in NLRP3-dependent but caspase-1 and IL-1*β* independent manner.

YopM has been reported previously to block caspase-1 activity. We then investigate the possible impact of the YopM C68A mutant on inflammasome activation. We first transfected WT YopM or its C68A mutant into BMDMs and use LPS+ATP or LPS+CTB as canonical/noncanonical NLRP3 inflammasome agonist. Both WT YopM and C68A overexpression inhibited caspase-1 activation (Figure 5g). In consistence, both ΔYopM/YopM and ΔYopM/C68A infection failed to activate caspase-1 cleavage (Figure 5e). Furthermore, caspase-1 activity analysis by colorimetric assay showed that both YopM and its C68A mutant could inhibit caspase-1 activity (Figure 5h). Taken together, these results indicated that the ability of YopM to block the formation of the mature inflammasome and inhibit caspase-1 activation does not require its E3 ligase activity.

### YopM induces necrotic cell death *in vivo*

**Figure 6:**
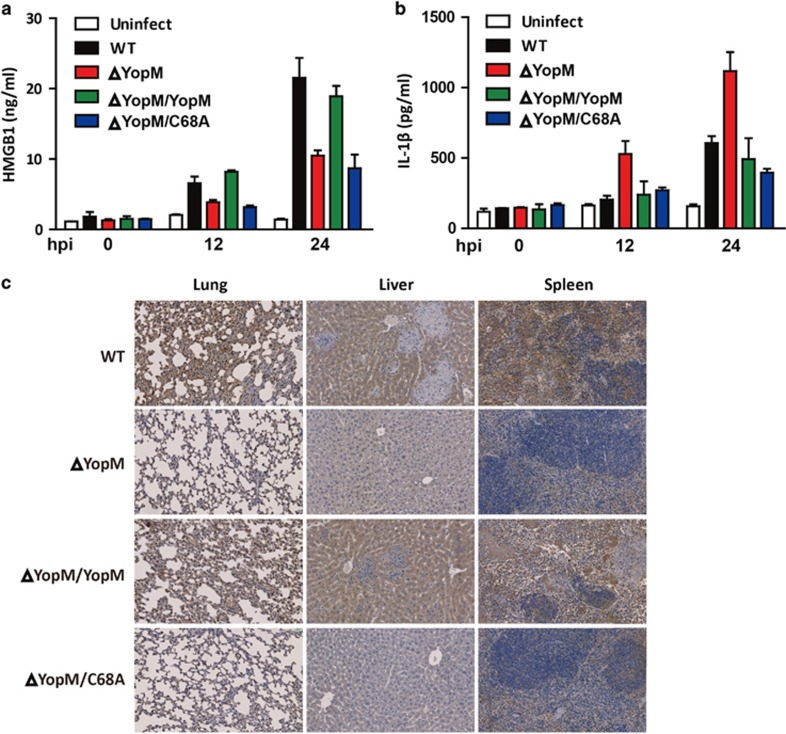
YopM E3 ligase activity contributes to NLRP3-mediated HMGB1 release and necrosis *in vivo*. (a-**b**) C57BL/6 mice were challenged by intravenous infection with 100 CFU WT, ΔYopM, ΔYopM/YopM, or ΔYopM/YopM C68A *Y. pestis*, serum were collected at 12 h and 24 h post infection and levels of HMGB1 (**a**) and IL-1*β* (**b**) were determined by ELISA. (**c**) Lung, liver, or spleen from mice infected with WT, ΔYopM, ΔYopM/YopM, or ΔYopM/YopM C68A strains for 3 days were subjected to immunohistochemical staining of NLRP3. All images are representative and were taken at × 40 magnification. Data shown are representative from two independent experiments (*n*=5 mice per strain)

To further delineate the role of YopM in necrotic cell death *in vivo*, we analyzed serum HMGB1 levels from mice infected with YopM and its mutants. As was shown in Figure 6a, WT and ΔYopM/YopM-infected mice displayed significantly increased serum HMGB1 levels as compared with the ΔYopM and ΔYopM/YopM C68A-infected mice with the infection time progressed (Figure 6a), while serum IL-1*β* levels were not markedly elevated in WT, ΔYopM/YopM, and ΔYopM/YopM C68A-infected mice (Figure 6b). Furthermore, significantly increased staining of NLRP3 in the inflammatory lesions of the lung, liver, and spleen (where bacteria are found) from the WT and ΔYopM/YopM infected mice as compared with ΔYopM and ΔYopM/YopM C68A was identified (Figures 6b and c). Therefore, YopM E3 ligase activity contributes to NLRP3-mediated HMGB1 release and necrosis *in vivo*.

### YopM E3 ligase activity contributes to bacterial virulence

**Figure 7:**
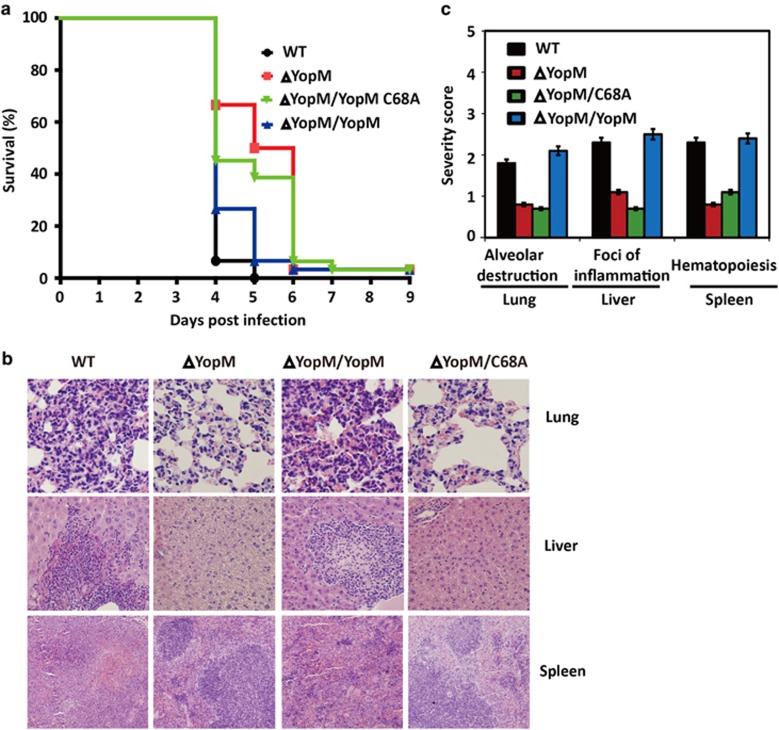
YopM E3 ligase activity is important for bacterial virulence. (**a**) Groups of 15 male and female mice were challenged by intravenous injection of 100 CFU WT, ΔYopM, ΔYopM/YopM, or ΔYopM/YopM C68A *Y. pestis* and monitored over 10 days for development of disease. Data shown were collected in two independent experiments, *n*=15 mice per group in each experiment. (**b**) Lung, liver, or spleen from mice infected with WT, ΔYopM, ΔYopM/YopM, or ΔYopM/YopM C68A strains for 3 days were subjected to histopathological analysis by staining with HE. Each micrograph shows an example of the pathology caused by each strain. Data shown are representative from two independent experiments (*n*=5 mice per strain). (**c**) Mean severity scores for histopathology of lungs, liver, and spleen from mice infected with different background of bacteria strains on day 3

HMGB1 releasing elicits a severe inflammatory response, leading to tissue injury, and lethal sepsis in several infectious diseases. The exacerbated HMGB1 release and cell necrosis induced by YopM E3 ligase activity indicates its potential role in pesitis virulence. After challenging mice by intravenous injection of WT, ΔYopM,ΔYopM/YopM, or ΔYopM/YopM C68A *Y. pestis*, Mice were more resistant to ΔYopM or ΔYopM/YopM C68A infection in overall survival assays (Figure 7a).

We then examined disease pathology by staining formalin fixed lungs, liver, and spleen with HE staining (Figure 7b). Total pathological severity scoring of lungs, liver, and spleen indicated a significantly increased inflammation, tissue injure and necrosis in WT and ΔYopM/YopM bacteria infected tissues, while the ΔYopM or ΔYopM/YopM C68A strain generally caused low levels of inflammation and necrosis (Figure 7c). Therefore, we conclude that YopM E3 ligase activity is essential for virulence.

## Discussion

During host–pathogen interactions, pathogens can release their PAMPs into the host cell cytosol by various means.^41^ NLRs and RIG-I-like receptors (RLRs) respond rapidly to PAMPs found within the host cell cytosol and trigger cell death.^42, 43^ Here we show that cytosolic detection of *Y. pestis*-derived T3SS effectors is a major mechanism of inducing necrosis, and this process requires signaling mainly through NLRP3. In addition, the E3 ligase activity of YopM functions as an essential signaling adaptor, linking the cytosolic detection of bacteria to the NLRP3-mediated necrotic cell death signaling pathway. Here we report evidence that YopM itself, a conservative molecule in *Y. pestis* species, is a unique simulator of NLRP3. The activity of YopM lies in its CLD motif, a conserved CXD motif located in N-terminal domain, which is responsible and crucial for the E3 activity of YopM. Our biochemical analysis identified a host protein, NLRP3, as the target of YopM. Although a number of NLRs were identified, we did not observe any effect of YopM on NLRP4 or NLRP5. Our results thus revealed a microbial product that is responsible for NLRP3-mediated necrotic cell death, in addition to the well-known bacterial secretion systems and other bacterial contaminants.

The expression of NLRP3 is tightly controlled to restrict its ability to directly recognize pathogens.^44^ Previous reports showed that the activation of inflammasome required both NLRP3 expression induction and NLRP3 inflammasome assembly.^45^ LPS is also known to induce NLRP3 mRNA and protein expression. This dual stimulation requirement may operate to prevent accidental or uncontrolled NLRP3 activation. However, the mechanisms governing NLRP3-induced necrosis have not been conclusively delineated. Here we found that YopM is a conservative *Y. pestis* product that can activate NLRP3 expression, YopM itself is sufficient for NLRP3-mediated necrosis as NLRP3 agonist by promoting its K63-linked ubiquitination, suggesting that cell necrosis may associated with K63-linked ubiquitination of NLRP3. Further characterization of the NLRP3 ubiquitination mechanism and the identification of the host NLRP3 ubiquitination enzymes would enhance our understanding of the mechanism of NLRP3 in necrosis signaling pathway.

Pathogens have been shown to use different strategies to modulate innate immune signaling pathway.^46, 47^ For example, Kaposi's sarcoma-associated herpes virus (KSHV) encodes a protein called Orf3 to interact with NLRP1, NLRP3, and NOD2 and to repress IL-1*β* production during KSHV infections.^48^ Salmonella is known to downregulate the expression of flagellin to avoid detection by the inflammasome.^49^ However, recent reports suggest that *S. typhimurium* activates caspase-1 directly through T3SS effector protein SopE.^50^ *Shigella* induced necrotic host cell death pathway requiring both NRLP3 and ASC, but caspase-1 independent.^51^ *Y. pestis* KIM-infected macrophages released HMGB1, and microscopic analysis revealed that necrotic cells contained active caspase-1, indicating that caspase-1 activation is associated with necrosis.^26^ Previous study indicated YopM is a pseudosubstrate inhibitor of caspase-1 activity, which as a potent antagonist of both caspase-1 activity and activation.^37^ Moreover, recent study indicated IQGAP1 is important for activation of caspase-1 in macrophages; YopM can also inhibit caspase-1 activation independently of a pseudosubstrate motif through IQGAP1 dependent pathway.^52^ Our results support previous data indicating that *Y. pestis* induced NLRP3-dependent yet caspase-1 independent necrosis, and contributes to bacterial virulence by inducing cell necrosis.

Pathogenic infection triggered the release of HMGB1, a proinflammatory factor released by necrotic cells. Once HMGB1 is released, several proinflammatory cytokines is induced and severe inflammatory response is elicited.^51^ Treatment with a neutralizing antibody of HMGB1 has been shown to significantly reduce inflammation and improve survival in animal models.^53^ We report that serum HMGB1 is increased during *Y. pestis* infection in mice. The release of HMGB1 elicited by *Y. pestis*-induced cell death further supports the use of HMGB1 antagonists to reduce mortality rate during *Y. pestis* infection.

Cathepsin B is a cysteine protease that is located in lysosomes and degrades in an acidic environment.^54^ Previous reports show that cathepsin B has some role in the process of NRLP3-mediated necrosis before inflammasome formation.^40^ Our data suggests that cathepsin B inhibitor CA-074-Me abrogated YopM induced NLRP3-dependent HMGB1 releases, indicating that cathepsin B is indispensable in this type of cell death.

Rapid bacterial replication in the absence of inflammation in the early phase transitions to a highly proinflammatory phase with increased inflammatory cytokine production and tissue damage.^55^ Host cell apoptosis decreases while pyroptosis or other forms of inflammatory cell death increase.^56^ *Y. pestis* initially replicate in the host without inducing inflammation by antagonize caspase-1-dependent pathway to enhance their virulence. An initial noninflammatory phase of *Y. pestis* replication is followed by inflammation. Here we proposed that YopM regulated necrotic cell death through NLRP3 in the later time during their infection. As opposed to the study by LaRock and Cookson showing that YopM deletion in *Y. pseudotuberculosis* YPIII (serogroup O:3) results in 100% survival, our host survival results suggested that YopM deficiency in *Y. pestis* was mainly associated with delayed mortality. YopM is composed of a variable number of 20 or 22 amino acid LRRs, which are known to mediate protein–protein interactions.^27^ Different *Yersinia* strains encode distinct YopM isoforms, with 15-LRR isoform in *Y. pseudotuberculosis* YPIII (serogroup O:3) and 13-LRR isoform in *Y. pestis* (91001) strains.^52^ The differences of YopM LRRs repeats between these two strains maybe the main cause of virulence difference. In addition, *Y. pseudotuberculosis* YPIII (serogroup O:3) was naturally deficient in YopT.^37^ YopM deficiency in YopT defective background may lead to varied phenotypes between *Y. pestis* and *Y. pseudotuberculosis* YPIII (serogroup O:3). Further investigation on the impact of YopM on *Y. pestis* induced necrosis would greater our understanding on the underestimated proinflammatory functions of YopM on plague pathogenesis.

## Materials and Methods

### Strains, plasmids and reagents

*Y. pestis* strain 91001 was used as the wild -type bacteria.^57^ The construction of *Y. pestis* strain 91001 with mutant YopM was carried out as described previously.^58, 59, 60^ The pACYC184 vector was used to construct the YopM or YopM C68A (the 68th cysteine residue was replaced by alanine) overexpressing strains. The resultant plasmids were introduced into *Y. pestis* ΔYopM strain. The YopM or YopM C68A coding sequence was amplified by PCR and cloned into pcDNA3-Flag, pCMV-Myc, and pCMV-HA vectors, respectively. Vectors and epitope tagging of Flag-tagged NLRP3 and their mutants were expressed by cloning the genes into the pcDNA3-based vector (Invitrogen, San Diego, CA, USA). Glutathione S-transferase (GST) fusion proteins were generated by expression in pGEX4T-1-based vectors (Amersham Biosciences Biotech, Inc., Uppsala, Sweden) in *Escherichia coli* BL21 (DE3). All plasmids were verified by restriction enzyme analysis and DNA sequencing. Cathepsin B inhibitor CA-074-Me was obtained from Sigma (C5857; Sigma, St Louis, MO, USA). YVAD-CHO was obtained from Enzo (ALX-260-027; Enzo, Farmingdale, NY, USA). Necrostatin-1 was obtained from Sigma (N9037).

### Cell culture and infection conditions

Human embryonic kidney (HEK293) cells were grown in Dulbecco's modified Eagle's medium (DMEM. Invitrogen) supplemented with 10% heat-inactivated fetal bovine serum (FBS, HyClone, Logan, UT, USA), 2 mM L-glutamine, 100 U/ml penicillin, and 100 mg/ml streptomycin. Transient transfections were performed with jetPRIME *in vitro* DNA and siRNA transfection reagent (Polyplus, NY, USA) following manufacturer's instructions. WT *Yersinia* were grown overnight with aeration in medium broth at 28–30 ºC, and ΔYopM/YopM *Yersinia* were grown with 10 *μ*g/ml kanamycin and amino benzyl penicillin. The ΔYopM/YopM and ΔYopM/YopM C68A RPMI1640 medium (without streptomycin and penicillin) was added with 20 *μ*g/ml Chloramphenicol 1 h before the infection. Bacteria were added to the cells at the indicated MOI and spun onto the cells at 1500 r.p.m. for 5 min. Cells were incubated at 37 °C for 0.5 h post infection followed by addition of 10 *μ*g/ml gentamicin.

### Bone marrow-derived macrophage (BMDM) isolation

Bone marrow cells from femur exudates of C57BL/6 were grown at 37 °C in a humidified incubator in RPMI 1640 medium (RPMI, Invitrogen) containing 10% FBS, 2 mM L-glutamine, 100 U/ml penicillin, 100 mg/ml streptomycin and 100 ng/ml mouse GM-CSF (Peprotech, Rocky Hill, NJ, USA) for 7 days. Differentiated BMDMs were replated into six-well dishes 18 h before infection.

### *In vivo* ubiquitination assay

HEK293 cells were co-transfected with plasmids expressing Flag-NLRP3, Myc-YopM, HA-tagged ubiquitin or HA-tagged K48-only ubiquitin or HA-tagged K63-only ubiquitin. Cells were immunoprecipitated with anti-Flag antibody. The polyubiquitination signal was detected using anti-HA of anti-NLRP3 antibodies.

For detection of endogenous ubiquitinated-NLRP3 following *Y. pestis* infection, cells were infected at MOI=20 and harvested for whole-cell extracts at the indicated times. Immunoprecipitation was performed on 1 *μ*g of protein with anti-NLRP3 antibody. After transfer, the membrane was denatured in 6 M guanidine-HCl solution (6 M guanidine-HCl, 20 mM Tris-HCl, pH 7.5, 1 mM phenylmethylsulfonyl fluoride, and 5 mM dithiothreitol) for 30 min at room temperature. The polyubiquitination signal was detected using an anti-ubiquitin monoclonal antibody (sc-166553, Santa Cruz Biotechnology, Dallas, TX, USA).

### *In vitro* ubiquitylation assay

*In vitro* ubiquitination assays (Enzo Ubiquitinylation Kit) were performed in 50 *μ*l reaction mixture containing reaction buffer (25 mM Tris-HCl at pH 7.5, 50 mM NaCl, 5 mM ATP, 10 mM MgCl_2_ and 0.1 mM DTT), 2.5 *μ*l E1, 5 *μ*l mixed E2, and 2.5 *μ*l ubiquitin purified in the presence or absence of GST-YopM or GST-YopM C68A at the indicated times. For *in vitro* NLRP3 ubiquitylation by YopM, reactions were performed as for autoubiquitination assays except that 1 *μ*g purified Flag-NLRP3 was included. The reactions were incubated at 37 °C for 1 h and stopped by the addition of 5 × Laemmli sample buffer.^61^

### Immunoblotting and Co-immunoprecipitation

Cell lysates were prepared in lysis buffer (50 mM Tris-HCl (pH 7.5), 1 mM phenylmethylsulfonyl fluoride, 1 mM dithiothreitol, 10 mM sodium fluoride, 10 mg/ml aprotinin, 10 mg/ml leupeptin, 150 mM NaCl, and 10 mg/ml pepstatin A) containing 1% NP-40. Soluble proteins were subjected to immunoprecipitation with anti-Flag or Anti-Myc Agarose Affinity Gel (Sigma-Aldrich) antibodies. An aliquot of the total lysates (5%, v/v) was included as control. Immunoblotting analysis was performed with anti-Myc, anti-HA, anti-Flag, anti-*α*-Tubulin (Sigma-Aldrich), anti-HMGB1 (ab92310, Abcam, Cambridge, UK), anti-caspase-1 (sc-514, Santa Cruz Biotechnology), anti-ASC (sc-271054, Santa Cruz), and anti-NLRP3 (AG-20B-0014-C100, Adipogen, San Diego, CA, USA) antibodies, respectively. The antigen-antibody complexes were visualized by chemiluminescence (5200, Tanon, Inc., Shanghai, China). The Band Analysis tools of Gel Image System software version 4.2 (Tanon) were used to select and determine the background-subtracted density of the bands in all the gels and blots. Level of protein was first normalized to respective tubulin control. The control (wild-type) condition was normalized to 1 and all other experimental conditions were compared with this.

### ELISA (enzyme-linked immunosorbent assay)

HMGB1 concentration was assayed by ELISA Kit (ST51011; Tecan, Männedorf, Switzerland) according to the manufacturer's instructions. IL-1*β* concentration was assayed by ELISA Kit (DKW12-2012-096; Dakewe, Shenzhen, Guangzhou, China) according to the manufacturer's instructions.

### Cycloheximide chase assay

HEK293 cells were transfected with plasmids expressing Flag-NLRP3 with or without Myc-YopM, Myc-YopMΔN, Myc-YopM C68A. Cells were collected by trypsinization after treatment with cycloheximide (10 *μ*M) for the indicated times and lysed for Immunoblotting analysis with anti-Flag antibody. The band intensity of the Immunoblotting result was measured by gel documentation system with the reading normalized as percentage of the initial NLRP3 level (level at time=0). The percentage was then plotted against time, and the half-life of the NLRP3 protein was calculated as the time required for degradation of 50% of the protein.

### Histopathological scoring

Lung, liver and spleen tissues were fixed in 10% phosphate-buffered formalin, dehydrated by a tissue processor and embedded in paraffin. Tissues were sectioned using a microtome (5 *μ*m) and H&E stained with an autostainer. Histological scoring of coded slides was evaluated by a single pathologist, blinded to the source of the slides, based on a modified system.

### Immunohistochemical (IHC) staining

IHC staining for NLRP3 was performed on the paraffin-embedded tissue microarray. The TMAs were performed on 5 *μ*m thick sections. Tissue slides were de-paraffinized and rehydrated through a graded alcohol series. Antigen retrieval was performed by placing the slides in 10 mM sodium citrate buffer (pH 6.0) and maintained at a sub-boiling temperature for 10 min. To block non-specific staining, the slides were immersed in 10% normal goat serum in phosphate-buffered saline (PBS) for 30 min. Then, primary antibody was used overnight at 4 °C in a humidified chamber. Slides were washed in PBS, followed by secondary antibody for 30 min at room temperature.

### Real-time RT-PCR

RNA was extracted with TRIzol reagent (Invitrogen). cDNA was synthesized with 1 *μ*g of total RNA using the Moloney murine leukemia virus reverse transcriptase (Transgen Biotech, Beijing, China) and random primers. Real-time PCR was performed using TransStart Green qPCR SuperMix (Transgen Biotech) and was analyzed on an ABI Prism 7500 analyzer (Applied Biosystems, Waltham, MA, USA). All real-time values were normalized to GAPDH in the same samples. Sequences of the primers are as follows: GAPDH forward primer: 5′-ACAACTTTGGTATCGTGGAAGG-3′ GAPDH reverse primer: 5′-GCCATCACGCCACAGTTTC-3′ NRLP3 forward primer: 5′- GATCTTCGCTGCGATCAACAG-3′ NRLP3 reverse primer: 5′- CGTGCATTATCTGAACCCCAC-3′.

### Cytotoxicity assay

Lactate dehydrogenase (LDH) content was measured in the aspirated media collected from cells infected after 2 or 6 h of infection. Samples were run in triplicate using the QuantiChrom kit (BioAssay Syst, Hayward, CA, USA) and the manufacturer's instructions. Percent cytotoxicity was assessed by comparing the amount of LDH in the supernatant to that recovered from control cells that were not infected following lysis by 0.1% triton X-100.

### Animals

Male and female wild-type C57BL/6 mice, ranging from 20–30 g were used for challenging experiments. During challenging with *Y. pestis* strains, mice were maintained in select agent authorized animal biosafety level 3 facilities at Beijing Institute of Biotechnology. All infected mice were monitored regularly by daily weighing and assignment of health scores.

### Ethics statement

All animals were handled in strict accordance to the 'Guide for the Care and Use of Laboratory Animals" and the "Principles for the Utilization and Care of Vertebrate Animals", and all animal work was approved by the Institutional Animal Care Committee of Beijing Institute of Biotechnology (protocol number 2012-001-13)

### Statistical analysis

All data were presented as average±standard error. Two-tailed Student's *t*-test was used for evaluating statistical significance between groups. **P<0.01 were considered to be statistically significant.
