# Supplementary material for: The Yersinia Type III secretion effector YopM Is an E3 ubiquitin ligase that induced necrotic cell death by targeting NLRP3
Source: Cell Death Dis. 2016 Dec 8;7(12):e2519–. doi: 10.1038/cddis.2016.413 (PMC5260993; doi:10.1038/cddis.2016.413)
Supplement: Supplementary information [file cddis2016413x1.docx]

**Supplemental Information**

The *Yersinia* Type III Secretion Effector YopM Is an E3 Ubiquitin Ligase that Induced Necrotic Cell Death by targeting NLRP3

Congwen Wei, Ying Wang, Xiang He, Zongmin Du, Ye Cao, Huiying Yang, Pengyu Zhou, Jiankang Chen, Penghao Wang, Zirui Zheng, Pingping Zhang, Yanhong Zhang, Shengli Ma, Ruifu Yang, Hui Zhong

Supplemental Experimental Procedures

**Yeast two-hybrid screening**

To perform the yeast two-hybrid screening, YopM(LRR domain) (amino acids 93–338) was inserted into the bait pGBKT7 vector for expression as a fusion protein with the Gal4 DNA binding domain (Gal4-BD). This plasmid was used to transform the AH109 yeast strain (Clontech, Palo Alto, CA), and Gal4-BD fused YopM(LRR domain) was used as bait in a mating strategy for the screening of human spleen cDNA library. The spleen cDNA library was inserted into the pGADT7 vector (Clontech, Palo Alto, CA) for expression as fusions with the Gal4 activation domain (Gal4-AD) and was maintained in the Y187 strain of yeast (Clontech, Palo Alto, CA). Transformed AH109 and Y187 yeast cells were mixed together for mating. Positive clones were selected on synthetic dropout medium lacking 4 nutrients (Leu/Trp/Ade/His). The blue colonies were kept, and the positive results were confirmed by repeating assays. cDNA plasmids isolated from positive colonies were introduced into Escherichia coli DH5α and sequenced. The sequences were analyzed with the BLAST program in NCBI.

**Caspase-1 activity colorimetric assay**

Recombinant caspase-1 (10 U; Enzo) was preincubated with GST-YopM or GST-YopM(C68A) for 5 min at 37℃ in assay buffer (Enzo). Caspase-1 substrate Ac-YVAD-pNA (Enzo) was added to 200 mM, and cleavage was detected by monitoring of absorbance at 405 nm (EnVision Multilabel Reader, Perkingelmer).

**FITC-Annexin V/PI assay**

BMDM cells were plated at a density of 1×10^6^ cells/ml medium in 50-mm diameter dishes. BMDMs infected with different Y. pestis strains including WT, ΔYopM, ΔYopM/YopM or ΔYopM/C68A. The cells were harvested from the culture dishes 24 h later by trypsinization. After washing twice with PBS, the cells were resuspended in 200 μl assay buffer. Then, 5 μl FITC-labeled Annexin V (Beyotime Biotechnology) and 5 μl PI were added. Upon incubation in the dark for 20 min at room temperature, the samples were analyzed with a FACS instrument (BD Calibur).

**Blot Imaging and Densitometric Analysis**

The Band Analysis tools of Gel Image System software version 4.2 (Tanon) were used to select and determine the background-subtracted density of the bands in all the gels and blots. Level of protein was first normalized to respective tubulin control. The control (wild type) condition was normalized to 1 and all other experimental conditions were compared to this.

**Figure S1. YopM protein associates with NLRP3, related to Figure 2.**

(A) AH109 was cotransformed with the plasmids as indicated. Positive interaction showed colony formation on synthetic medium lacking tryptophan, leucine, adenine, and histidine.

(B) HEK293 cells were co-transfected with Flag-NLRP3 and Myc-YopM expression plasmids or Myc-vector, and anti-Myc Agarose Affinity Gel or IgG agarose immunoprecipitates were analyzed by immunoblotting with anti-Myc or anti-Flag antibody.

(C) HEK293 cells were co-transfected with Flag-NLRP3 and Myc-YopM (KIM) expression plasmids or Flag-vector, and anti-Flag M2 Affinity Gel or IgG agarose immunoprecipitates were analyzed by immunoblotting with anti-Myc or anti-Flag antibody.

Cell-based studies were performed at least three times independently with comparable results.

**Figure S2. YopM mediates the stabilization of NLRP3, related to Figure 4.**

(A) Immunoblotting analysis of TBK1, ASC, pro-caspase-1, NLRP4, and NLRP5 levels in HEK293 cells transfected with increasing doses of plasmid for YopM and other indicated plasmids. α-tubulin was used as equal loading control.

Cell-based studies were performed at least three times independently with comparable results.

**Figure S3. YopM induces NLRP3-dependent necrosis *in vitro*, related to Figure 5.**

(A) Flow cytometry for BMDM cells stained with annexin V and propidium iodide following 24 h infection with indicated *Y. pestis* strains.

(B) BMDMs cells were treated with or without caspase-1 specific inhibitor YVAD-CHO, Cathepsin B specific inhibitor Ca-074-Me or RIP1 specific inhibitor necrostatin-1 prior to infection. BMDMs were infected with *Y. pestis*, supernatants were collected at 24 h after transfection and analyzed for LDH release.

(C) BMDMs cells were treated with or without caspase-1 specific inhibitor YVAD-CHO, Cathepsin B specific inhibitor Ca-074-Me or RIP1 specific inhibitor necrostatin-1 and then transfected with expression plasmids encoding for Flag-YopM or its mutants. Cell extracts and supernatants were prepared at 24 h. The whole cell lysates were analyzed by immunoblotting with anti-NLRP3 Abs. The supernatants were analyzed by immunoblotting with anti-HMGB1 Abs. α-Tubulin was used as equal loading control.

**Table S1. Local sequence alignment of *Yersinia Pestis* 91001 YopM with other bacterial effectors or YopM from other strains. Conserved residues are marked in red.**
