# Supplementary figures and images for: The Yersinia Type III secretion effector YopM Is an E3 ubiquitin ligase that induced necrotic cell death by targeting NLRP3
Source: Cell Death Dis. 2016 Dec 8;7(12):e2519–. doi: 10.1038/cddis.2016.413 (PMC5260993; doi:10.1038/cddis.2016.413)

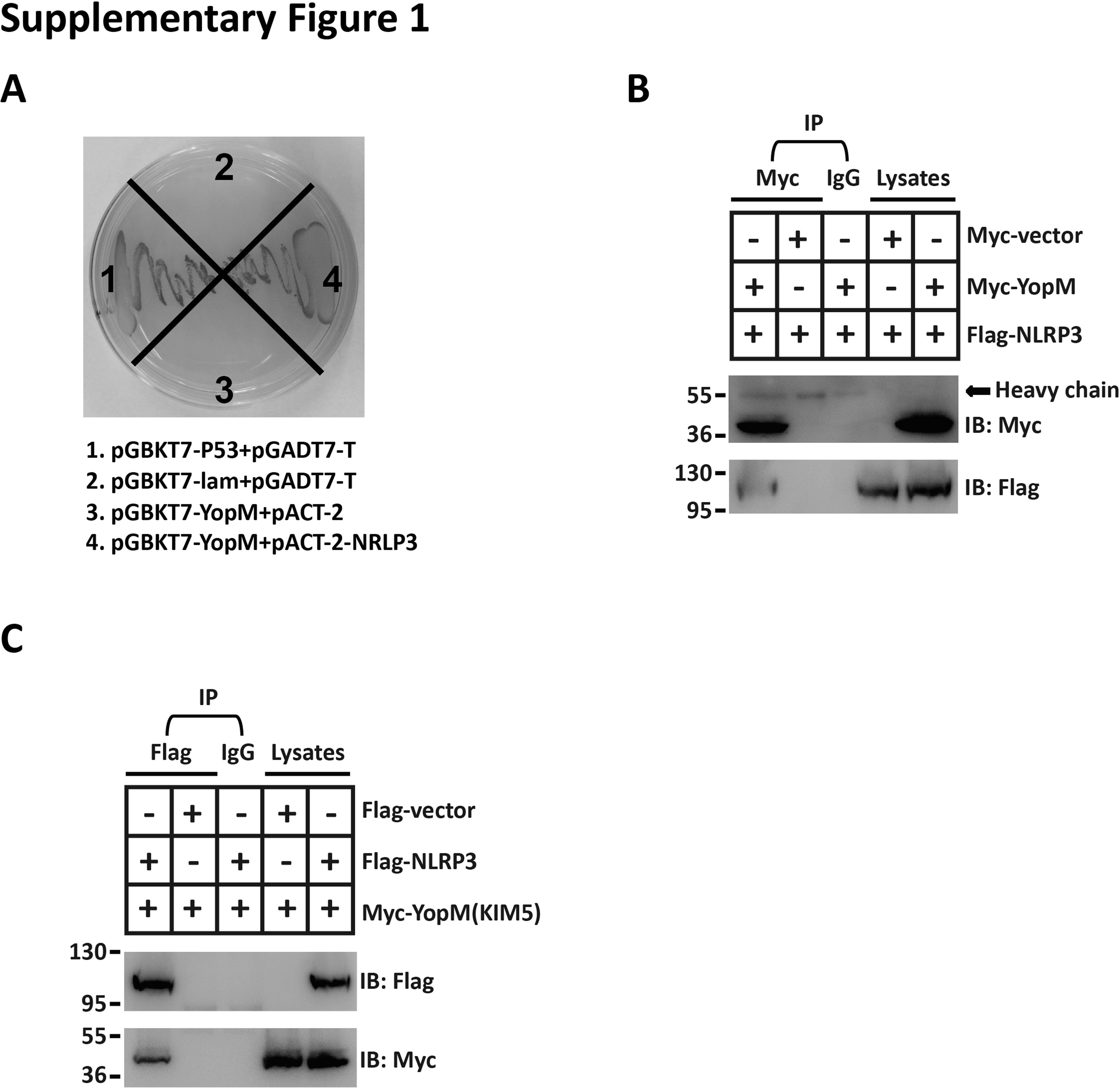

Supplement: Supplementary Figure 1 [file cddis2016413x2.tif]

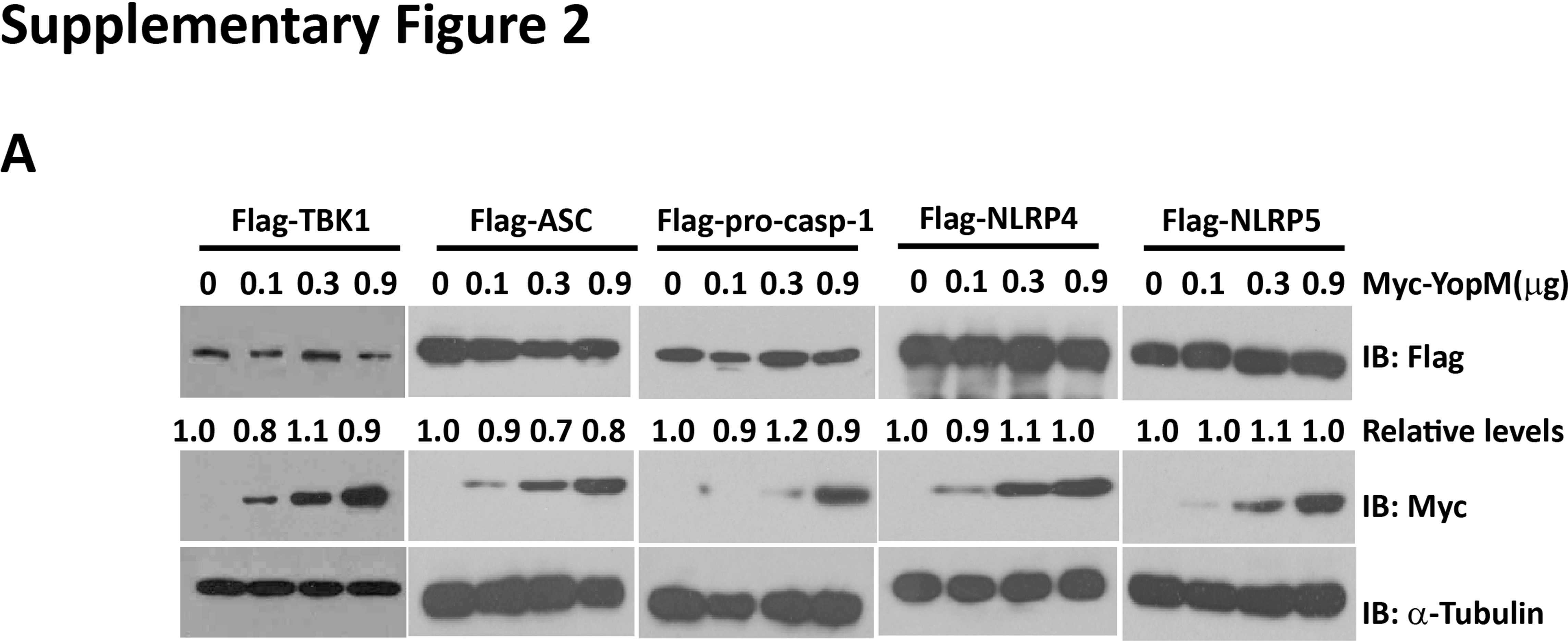

Supplement: Supplementary Figure 2 [file cddis2016413x3.tif]

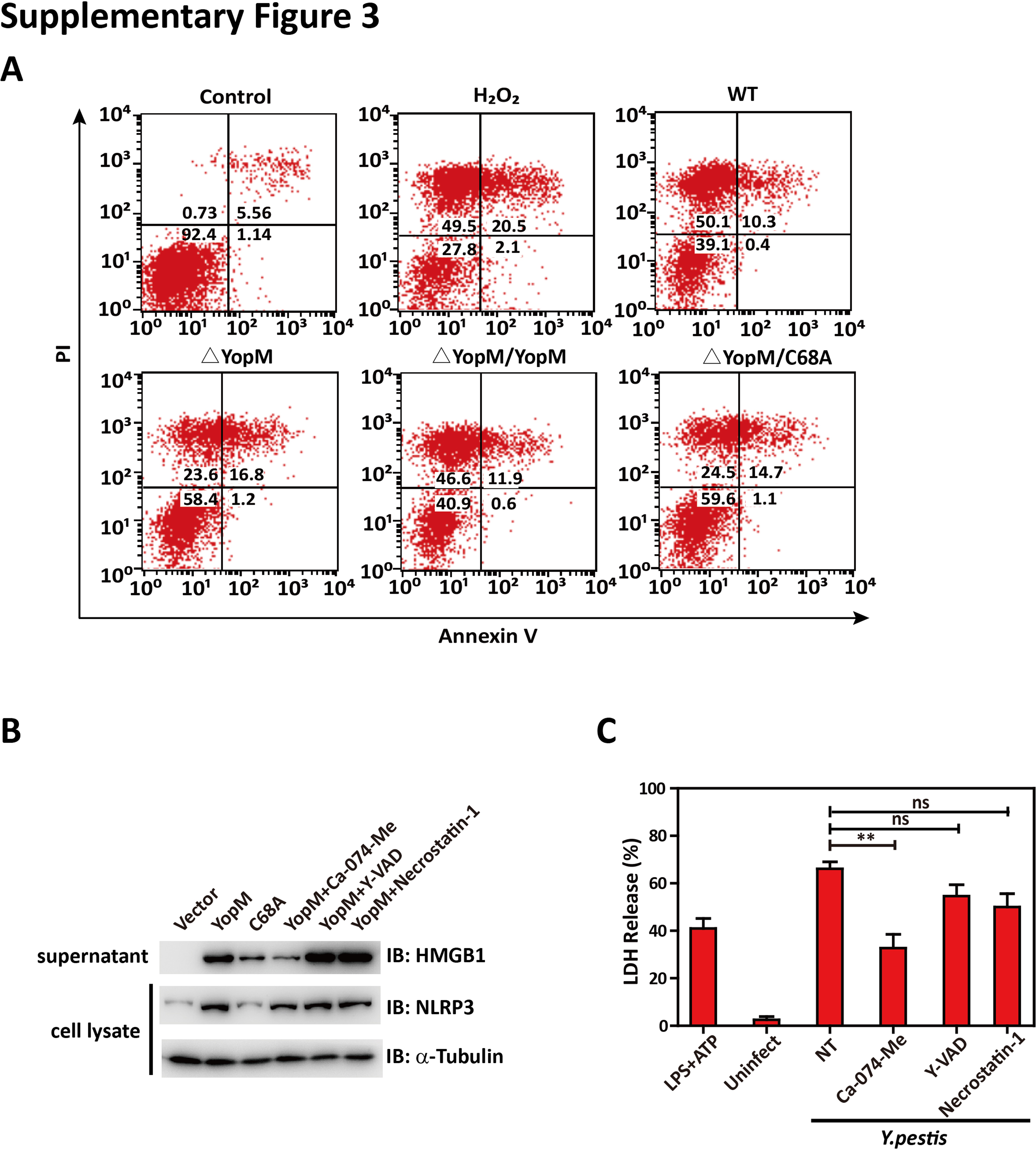

Supplement: Supplementary Figure 3 [file cddis2016413x4.tif]

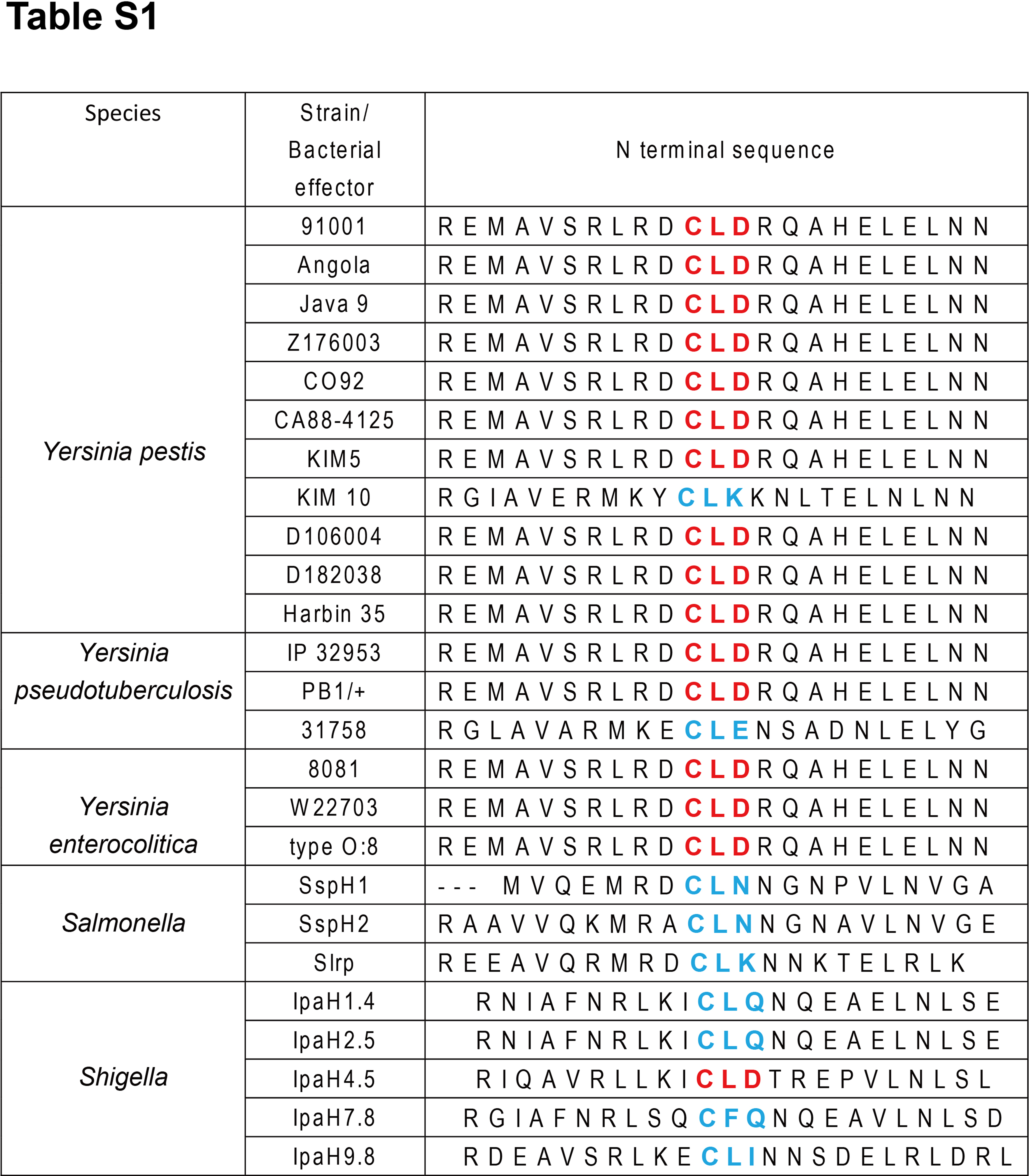

Supplement: Supplementary Table 1 [file cddis2016413x5.tif]
